# Rapamycin Loaded Solid Lipid Nanoparticles as a New Tool to Deliver mTOR Inhibitors: Formulation and *in Vitro* Characterization

**DOI:** 10.3390/nano6050087

**Published:** 2016-05-09

**Authors:** Alice Polchi, Alessandro Magini, Jarosław Mazuryk, Brunella Tancini, Jacek Gapiński, Adam Patkowski, Stefano Giovagnoli, Carla Emiliani

**Affiliations:** 1 Department of Chemistry, Biology and Biotechnology, University of Perugia, Via del Giochetto, 06126 Perugia, Italy; alicepolchi@virgilio.it (A.P.); alessandro.magini@unipg.it (A.M.); brunella.tancini@unipg.it (B.T.); carla.emiliani@unipg.it (C.E.); 2 NanoBioMedical Centre, Faculty of Physics, Adam Mickiewicz University in Poznań, 61614 Poznań, Poland; jmaz@amu.edu.pl (J.M.); gapinski@amu.edu.pl (J.G.); patkowsk@amu.edu.pl (A.P.); 3 Molecular Biophysics Division, Faculty of Physics, Adam Mickiewicz University in Poznań, 61614 Poznań, Poland; 4 Department of Pharmaceutical Sciences, University of Perugia, 06123 Perugia, Italy

**Keywords:** rapamycin, solid lipid nanoparticles, drug delivery, formulation, SH-SY5Y neuroblastoma cells

## Abstract

Recently, the use of mammalian target of rapamycin (mTOR) inhibitors, in particular rapamycin (Rp), has been suggested to improve the treatment of neurodegenerative diseases. However, as Rp is a strong immunosuppressant, specific delivery to the brain has been postulated to avoid systemic exposure. In this work, we fabricated new Rp loaded solid lipid nanoparticles (Rp-SLN) stabilized with polysorbate 80 (PS80), comparing two different methods and lipids. The formulations were characterized by differential scanning calorimetry (DSC), nuclear magnetic resonance (NMR), wide angle X-ray scattering (WAXS), cryo-transmission electron microscopy (cryo-TEM), dynamic light scattering (DLS) and particle tracking. *In vitro* release and short-term stability were assessed. Biological behavior of Rp-SLN was tested in SH-SY5Y neuroblastoma cells. The inhibition of mTOR complex 1 (mTORC1) was evaluated over time by a pulse-chase study compared to free Rp and Rp nanocrystals. Compritol Rp-SLN resulted more stable and possessing proper size and surface properties with respect to cetyl palmitate Rp-SLN. Rapamycin was entrapped in an amorphous form in the solid lipid matrix that showed partial crystallinity with stable Lβ, sub-Lα and Lβ′ arrangements. PS80 was stably anchored on particle surface. No drug release was observed over 24 h and Rp-SLN had a higher cell uptake and a more sustained effect over a week. The mTORC1 inhibition was higher with Rp-SLN. Overall, compritol Rp-SLN show suitable characteristics and stability to be considered for further investigation as Rp brain delivery system.

## 1. Introduction

Nanoparticles represent one of the most innovative non-invasive approaches for the delivery and targeting of drugs and pharmacologically active substances. The drug-loaded nanoparticles provide several advantages with respect to the free drugs, such as protection against degradation and promote a suitable, selective and specific targeted therapy and the increase in the patient compliance [1]. Drug delivery across the blood-brain barrier is a major limitation in the treatment of central nervous system disorders. The presence of the barrier excludes many compounds from reaching the suitable concentration in the brain tissue to produce the desired therapeutic effect, unless systemic side effects. Thus, the development of new treatment strategy for neurodegenerative diseases is a therapeutic challenge. Among nanoparticulate carriers, solid lipid nanoparticles (SLN) are promising candidates to treat neurodegenerative disorders to their brain targeting potential [2]. SLN are colloidal systems made of a homogenous solid lipid matrix, commonly stabilized with surfactants. These nanocarriers show high safety, scalability as well as a specific brain uptake mechanism [3]. SLN are known to favor specific adsorption of apolipoproteins on the particle surface when functionalized, in particular with polysorbate 80 (PS80), and this allows binding to the relative receptors on the blood-brain barrier, which triggers transcytosis [4,5].

In this work, we report for the first time the development of rapamycin (Rp) SLN formulations for future application in specific Rp brain delivery for the treatment of neurodegenerative disorders.

To our knowledge, this is the first lipid Rp colloidal formulation, whereas only lipid microparticles for oral delivery are documented in literature and Rp colloidal nanocrystals dispersions have been employed in Rapamune tablets [6,7].

Rapamycin is a well-known macrolide isolated from Streptomyces hygroscopicus in the 1970s [8]. Early studies on *Saccharomyces cerevisiae* identified FK506-binding protein 12 (FKBP12) and the TOR1 and TOR2 proteins (Target of Rapamycin) as the targets of rapamycin [9]. Shortly after, the mammalian target of rapamycin (mTOR), the ortholog of the yeast TOR1 and 2 proteins, was established [10,11]. The mammalian target of rapamycin is the catalytic subunit of mTORC1 and mTORC2 [12,13]. Rapamycin inhibits mainly mTORC1 through the association with its intracellular receptor FKBP12 [14].

The mTOR senses cellular nutrient, oxygen and energy levels and is strongly regulated by upstream pathways, including insulin, growth factors and amino acids. The mTOR pathway has been found deregulated in Alzheimer’s disease (AD). The mTOR signaling has been intertwined to the presence of soluble amyloid beta (Aβ) and tau proteins and therefore to the formation of Aβ plaques and neurofibrillary tangles. Findings demonstrated that the mTOR signaling is hyperactive in AD brains [15,16]. In addition, the mTOR activity and the expression of p70S6K, a downstream target of mTOR highly expressed in neurons developing neurofibrillary tangles, have been shown to be significantly increased in the cortex and hippocampus of AD animal models [17,18,19,20,21].

The mTOR activity in neurodegeneration is therefore a key factor even considering the fact that disruptions in autophagy may be a potential source of pathogenesis in protein misfolding diseases [22,23,24,25,26,27,28]. Treatment with Rp and related mTOR inhibitors could be beneficial in slowing down and controlling progression of neurodegenerative diseases. In 2010, the Oddo’s group showed the effects of Rp oral administration on an AD mouse model. The treatment demonstrated rescue of learning deficit and reduction of the intraneural Aβ [19].

Systemic administration of Rp, albeit beneficial, produces a number of side effects that may impair the therapeutic efficacy. Rapamycin is distributed extensively in many organs, brain included, and it shows a very long half-life (>60 h). However, in mammals, rapamycin displays a strong immunosuppressant effect when given systemically [29,30]. Such effect limits the administration of the drug on long-term or semi-chronic therapeutic regimen. For this reason, the intrathecal administration of mTOR inhibitors has been proposed to treat neurodegeneration, neuroinflammation and related cancer diseases [31]. However, this approach suffers from high invasiveness that reduces compliance and feasibility.

The use of nanoparticles for specific brain-targeted delivery of Rp and, in general, mTOR inhibitors, may reduce the systemic exposure and the strong immunosuppression effect by preserving compliance.

Therefore, we formulated Rp loaded SLN (Rp-SLN) and characterized them thoroughly *in vitro*. The efficacy of the Rp-SLN treatment was evaluated on SH-SY5Y cells, a human neuroblastoma derived cell line widely used as *in vitro* model of neuronal function [32]. This work was intended to establish a starting platform for the development of an alternative treatment of neurodegeneration.

## 2. Results and Discussion

### 2.1. Rp-SLN Physicochemical Characterization

#### 2.1.1. Effect of Different Preparation Methods and Lipids on SLN Properties and Short-Term Stability

The different SLN batches produced using the Ultrasound-Assisted Emulsion/evaporation (UAEe) and cold High Pressure Homogenization/evaporation (cHPHe) methods are displayed in Table 1. The two lipids employed provided different SLN characteristics in terms of size and drug entrapment. Being Rp hydrophobic and almost insoluble in water, it was entrapped successfully either in compritol or cetyl palmitate SLN. The cHPHe method provided the highest encapsulation efficiency (EE) and drug content (DC). The best EE and DC values ranged between 70%–89% and 7%–17% *w*/*w* (preparations #3–5, Table 1), respectively, and, in spite of a higher DC, increasing the theoretical loading from 10% to 20% *w*/*w* did not produce any EE improvement (preparation #5, Table 1). However, the SLN were large and sometimes aggregated, with mean hydrodynamic diameter (MHD) between 120 and 750 nm and polydispersity index (PI) > 0.4. This behavior may be ascribable to the high shear and cavitation produced at 1500 bars during homogenization cycles that can induce the formation of ultrafine droplets prone to aggregation and fusion upon the solvent evaporation step. On the other hand, the UAEe method produced smaller SLN with MHD between 70 and 250 nm. Albeit lower, %DC and %EE were still acceptable with best values around 4.4% and 45% *w*/*w*, respectively.

The better SLN characteristics obtained by the UAEe method is likely due to the difference in the way the shear is applied on the oil/water emulsion. While in the case of cHPHe the solvent evaporation occurs after homogenization, in the UAEe method the shear is simultaneous to the evaporation step and this prevents, at least partially, from any fusion and aggregation processes. 

Regrettably, such advantage in the case of particle size control, reduced consistently the entrapment efficiency of the UAEe method. Being Rp rather soluble in chloroform, such efficiency drop could be explained by considering two phenomena: (1) the drug outward diffusion upon ultrasound assisted emulsification that can be ascribed to higher local temperatures (final temperature > 50 °C) before lipid hardening and shell formation; (2) the cavitation forces characteristic of the ultrasound shear that may lead to local transient formation of ultrafine droplets, that exponentially increase the surface area of the chloroform/water interface and, thereby, the likelihood of the drug diffusion into the continuous phase. Upon cooling, the drug molecules, that diffused outside, could recrystallize and precipitate as it was observed in some of the preparations. This is the reason why loading was not increased above 10% *w*/*w*, since additional drug would have resulted only in more precipitation.

The zeta potential (Pz) values of Rp-SLN and blank SLN suggest that the higher Rp content obtained with the cHPHe method, may be the consequence of a higher localization of the drug on the SLN surface. In fact, Pz values are significantly lower, on average, than those of SLN fabricated by the UAEe method, and many of them are close to zero. This outcome imply two important consequences: (i) an apparent drug loss and fast release; and (ii) higher tendency to aggregation.

Differences in aggregation tendency were observed between compritol and cetyl palmitate, especially in the UAEe method. This behavior can be explained considering that cetyl palmitate is a softer lipid (T_m_ = 54 °C) than compritol and, due to the continuous sonication produced extra heating of the emulsion that reached 42 °C at the end of the evaporation process, the resulting SLN remained soft and, thereby, prone to clumping into larger particles.

This also explains why a 2% *w*/*v* PS80 concentration was required to stabilize cetyl palmitate SLN in the UAEe method, meanwhile a 1% *v*/*v* PS80 was enough in the cHPHe method, where the evaporation was conducted at room temperature. In the cHPHe method however, as pointed out above, the SLN were generally larger even due to crystallization of unentrapped Rp, either at fast or slow evaporation rates.

**Figure 1:**
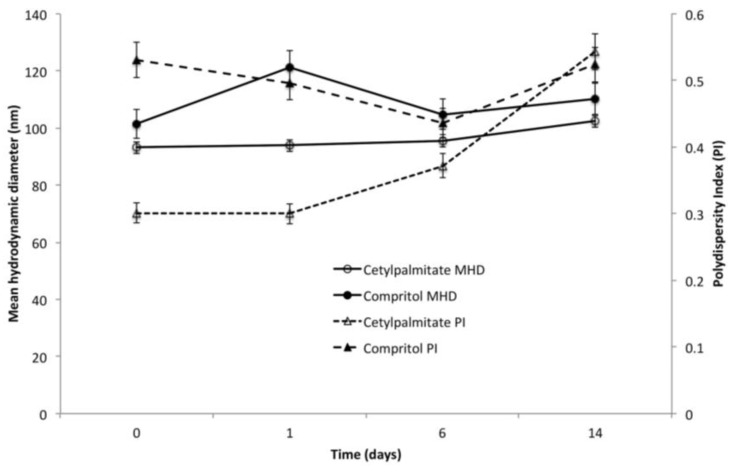
Hydrodynamic size and polydispersity change over time for Rp loaded compritol and cetyl palmitate SLN.

For all the above reasons, the UAEe method was preferred and the batches #2, for compritol SLN, and #6, for cetyl palmitate SLN, were chosen to check the short-term stability issues. Figure 1 depicts the MHD and polydispersity index (PI) profiles for the two above batches at 4 °C over about 14 days of incubation.

While no changes in %DC were observed for both lipids (data not shown), cetyl palmitate SLN showed a progressive increase of PI that raised from about 0.30 to 0.55 as a result of aggregation of smaller particles. This aggregation caused only a slight increase in MHD. In turn, compritol SLN remained unchanged, either in size or PI, indicating a higher stability compared to cetyl palmitate SLN. For this reason, compritol SLN were selected for the studies thereafter. 

#### 2.1.2. Morphology and Particle Tracking Analysis

**Figure 2:**
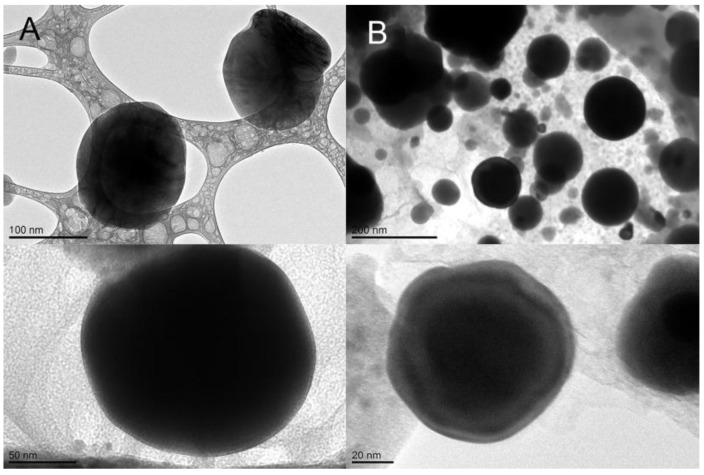
Cryo-transmission electron microscopy (cryo-TEM) images of blank (**A**) and Rp-SLN (**B**). Magnification 180,000× and 200,000×.

Compritol Rp-SLN were analyzed for morphology and dimensional populations. Figure 2 presents the results of cryo-TEM analysis, showing regular and spherical particles and marked roundness of blank and Rp-SLN. No particular differences caused by the Rp loading were observed since the surfaces seem to be rather regular as well.

**Figure 3:**
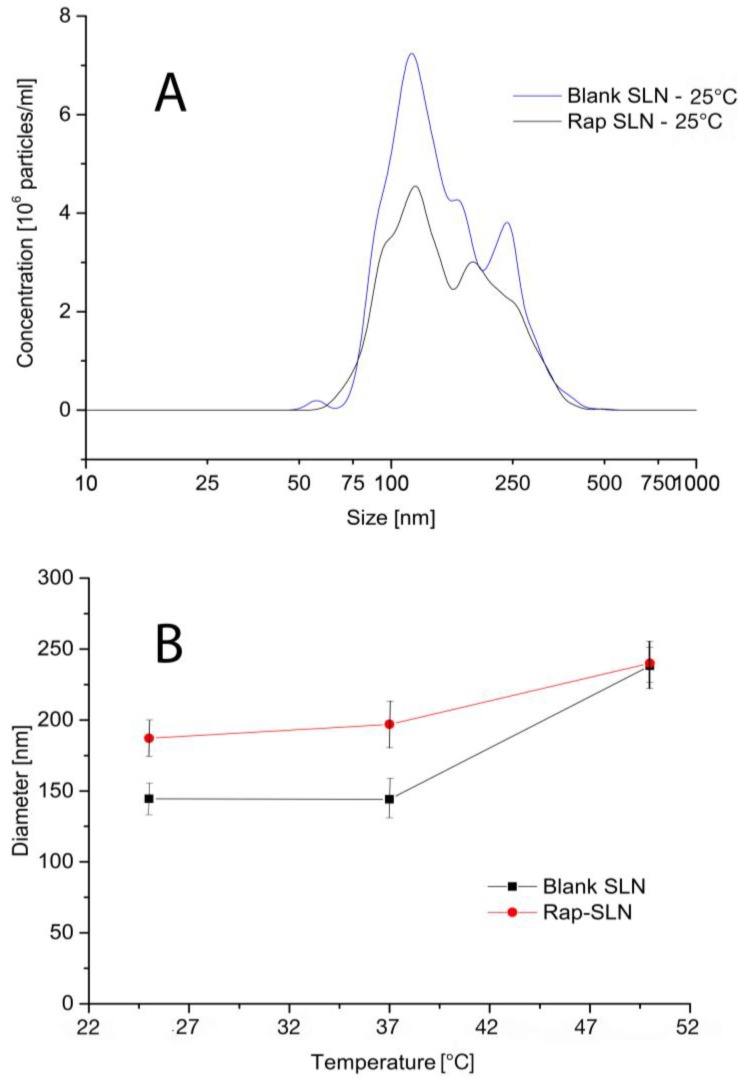
Particle tracking: dimensional profiles of blank and Rp-SLN at 25 °C (**A**) and an effect of temperature on the mean MHD of blank and Rp-SLN (**B**).

Particle tracking analysis was performed to highlight the presence of subpopulations and to evaluate the effect of temperature on the size of compritol Rp-SLN. Figure 3 shows the size distributions of blank and Rp-SLN acquired at 25 °C, as well as the hydrodynamic size change with temperature (Figure 3B). The size distributions of both blank and Rp-SLN spread from 50 nm to 500 nm and exhibit the presence of two main populations at 100 and 250 nm for blank SLN and 100 and 200–220 nm for Rp-SLN. These populations occurred rather consistent with temperature. The temperature effect on the mean size is reported in Figure 3B. The mean size was stable for both blank and Rp-SLN up to 37 °C, which indicates good stability at physiological temperature, and only at 50 °C the size increased for both nanoformulations to 240–250 nm. This observation can be associated to an increase in respective diffusion coefficients. The higher mobility of the particles raises the probability of collisions, interactions and coalescence leading to larger populations. It is worth to mention that temperature of 50 °C is much below the melting point of compritol (65–77 °C), and no physical changes in the SLN structure were observed upon the measurement.

#### 2.1.3. Thermal Analysis of Rp-SLN

**Figure 4:**
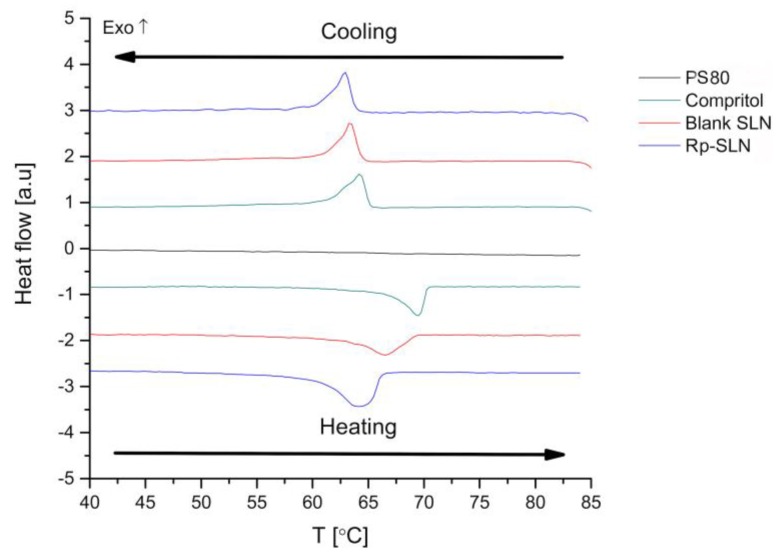
Heating and cooling ramps of blank and Rp-SLN compared to bulk compritol and PS80.

Thermograms in Figure 4 show first-order endotherms (upon heating) and exotherms (upon cooling) recorded for 1% (*v*/*v*) aqueous solution of PS80, pure compritol and both blank SLN and Rp-SLN. The bulk lipid and both SLN formulations promptly regained the solid form after the nanoparticles preparation and melting. In case of both SLN samples, endotherms were slightly broader compared to bulk compritol, displaying accentuated asymmetry due to the presence of at least two bands between 60 and 70 °C. These two bands may be assigned to the main sub-Lα lattice of dibehenin chains, and a minor contribution of the triglyceride sub-Lα or Lβ′ arrangements [33].

The absolute values of ΔH_m_ and ΔH_c_ are remarkably lower in the case of blank and loaded SLN in comparison to the bulk lipid (Table 2). Similarly, both melting and recrystallization points are shifted to lower temperatures for SLN when compared to pure compritol, what may suggest a lower order of crystallinity of the SLN lipid matrix. The recrystallization index (RI) values of 33.3% and 40.5% obtained for blank and Rp-SLN, respectively, (Table 2) show clearly that formation of a full crystal lattice after the SLN formulation is impeded, probably due to fast solidification and the presence of PS80 molecules on the particle surface. In turn, the relatively small supercooling effect (SC) values (3.6 °C, 0.8 °C, respectively), indicate the absence of supercooled or eutectic melts [34].

The effect of PS80 on SLN crystallinity is consistent with the literature [35], showing that stabilization of compritol SLN with PS80 and cholic acid mixture reduces the crystallinity down to 64%. The use of molecularly different species leads to defects in the internal arrangement of a crystal lattice. Moreover, nanosized particles possess a higher surface area and surface-to-volume ratio, hence a lower amount of energy is required for the disruption of SLN matrix than in case of pure crystalline lipid structure [36].

The Rp loading increases the ΔH_m_ and ΔH_c_ values in comparison to blank SLN, whereas reduces the transition points. This influence on the melting point can be explained by the fact that the Rp inclusion into the lipid matrix reduces the crystallinity order, whereas the difference in melting enthalpies may result from the larger size of Rp-SLN, as shown above. These observations correlate with the effect observed for other drugs on compritol SLN [37,38]. In addition, although the drug molecules are expected to distribute homogenously in the lipid matrix in an amorphous form, the Rp aggregation over time caused by lipid polymorphism cannot be excluded.

#### 2.1.4. NMR Analysis of Rp-SLN

**Figure 5:**
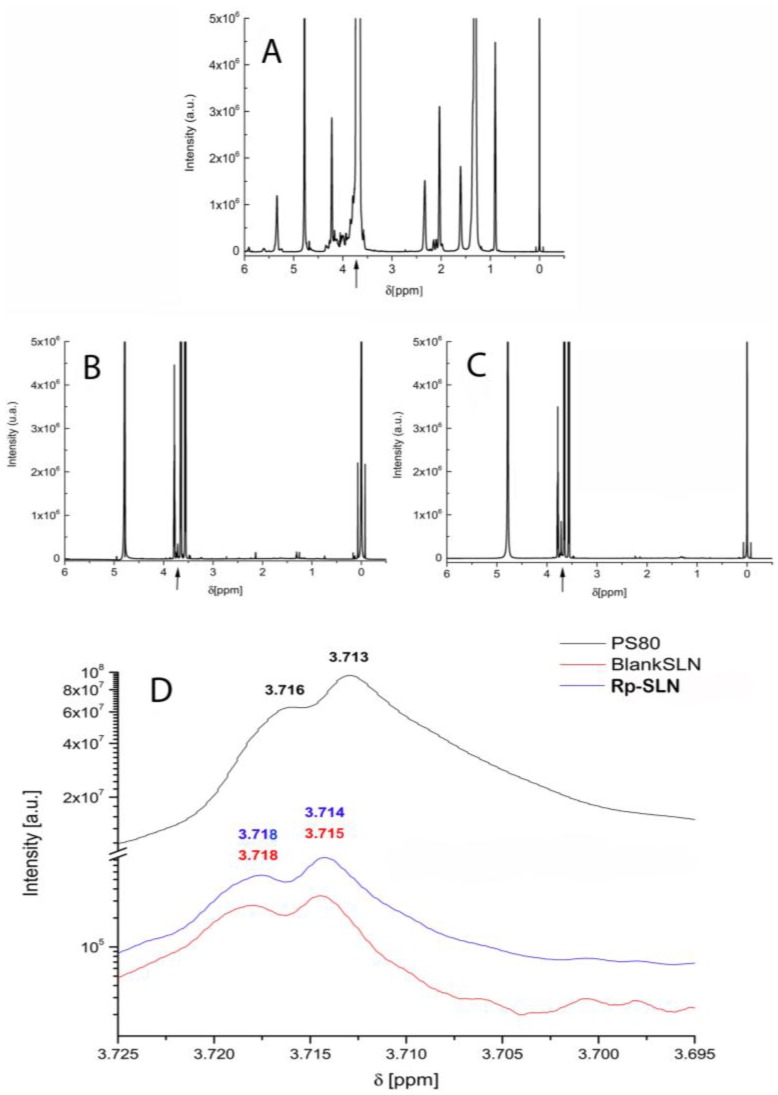
Proton nuclear magnetic resonance spectroscopy (^1^H NMR) spectra of (**A**) PS80; (**B**) blank and (**C**) Rp-SLN all prepared in D_2_O, were submitted to an external magnetic field of 18.8 T and ^1^H resonance frequency of 800 MHz. The arrows indicate the area magnified in (**D**) corresponding to the PS80-derived oxyethylene moiety signals (about δ = 3.7) to highlight the slight shift occurring in SLN compared to pure PS80.

The ^1^H NMR measurements of Rp in deuterated DMSO (DMSO-d_6_) were adjusted to allow the spectra comparison with the respective Rp-SLN profile in D_2_O. Rp signals were not detectable in SLN (Figure 5A–C). This may be a consequence of either low drug concentration or signal suppression arisen from the constraint of Rp molecules in the solid matrix [37,39,40,41,42]. In any case, the Rp loading produced only slight broadening of the characteristic compritol lipid signals between 0.7–1.6 ppm and 1.9–3.0 ppm for blank and loaded SLN. The same effect was observed for the PS80-specific oxyethylene and glyceride peaks between 3.0 and 4.0 ppm. These data suggest that Rp was entrapped physically in the lipid matrix with no chemical interaction with lipid molecules.

Regarding the PS80 contribution, a significant decrease of the broad band intensity at 3.72 ppm is observed in case of the oxyethylene groups of PS80 in the SLN, when compared to the signal of the pure surfactant (Figure 5d). Changes in shape and intensity of this hydrophilic group support interaction between PS80-stabilized SLN with water environment. The anchoring of PS80 on the surface of SLN decreases noticeably the magnitude, but not the shape, of these signals, since the hydrophilic part remains in constant interaction with the aqueous phase. The evidence of such PS80 anchoring on the SLN surface is provided by the disappearance of the olefinic proton signals at 5–6.5 ppm in both PS80-SLN spectra, that is characteristic for the intercalation of the oleic chain in the lipid matrix [40,43].

#### 2.1.5. Wide-Angle X-ray Scattering Analysis

**Figure 6:**
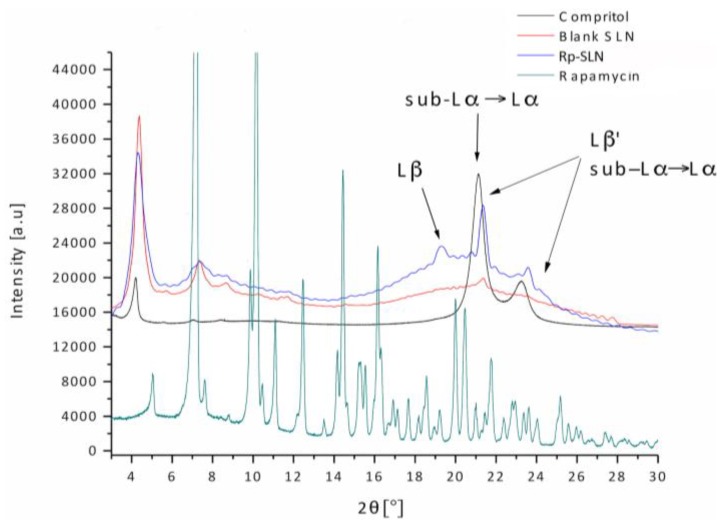
WAXS profiles of pure Rp, compritol and blank and Rp-SLN. Arrows indicate the signals corresponding to the polymorphs observed for blank, Rp-SLN and bulk lipid.

WAXS analysis was performed to identify the lipid polymorphic state on Rp-SLN compared to blank SLN and compritol. This step is important to forecast possible tendency of lipid rearrangement that may cause the drug losses and particle aggregation upon storage. Compritol consists of a mixture of mono- (20%), di- (50%) and tribehenin (30%), thus displays a complex chemical and crystalline composition. The WAXS signals and latiice spacings *d* values of compritol, Rp and both blank and Rp-loaded SLN, are shown in Table 3 and Figure 6. In the 2θ = 4°–13° and 17°–25° intervals the bulk lipid and SLN share the same Bragg distances reflecting a lamellae periodic structure [44]. Nevertheless, significant peak shifts and changes in intensity point out the presence of lipid polymorphs, commonly characterized by different lattice spacings: pseudo-hexagonal sub-Lα (0.415–0.42 nm), hexagonal Lα (0.415–0.42 nm), orthorhombic perpendicular Lβ′ (0.376–0.385 nm and 0.42 nm) and triclinic parallel Lβ (0.45–0.46 nm) [33,45].

Compritol is characterized by the *d* values of approximately 0.42 nm (2θ = 21.14°) and 0.38 nm (2θ = 23.25°), which are attributed to sub-Lα, Lα and Lβ′ polymorphs (Figure 6). An extent of non-stable sub-Lα and metastable Lβ′ forms is also characteristic for SLN and, in particular, for blank SLN. The diffractograms show a reduced intensity of signals in the 2θ range of 17°–25° especially for blank SLN. However, the signals detectable for blank and Rp-SLN confirm the co-existence of a metastable Lβ′ and an additional stable Lβ polymorph (*d* = 0.455 nm, 2θ = 19.48°; *d* = 0.459 nm, 2θ = 19.30°, Table 3). A decrease in intensity and line-broadening of the SLN peaks indicate the reduced crystallinity of the lipid matrix, mostly caused either by the process of preparation or the drug loading. In particular, the diffraction patterns reveal that the entrapment of Rp markedly influenced the ordering of Rp-SLN with respect to blank SLN, which may imply a difference in recrystallization rate.

The WAXS signals of Rp (Figure 6) are not detectable in Rp-SLN, which suggests the incorporation of the drug into SLN in an amorphous form. Owing to the hydrophobic nature of the drug and the mild changes in *d*-spacing and intensity compared to bulk lipid, Rp is either uniformly distributed in the matrix or deposited in a form of very small crystallites. The presence of imperfect sub-Lα and Lβ′ lattices in the lipid matrix facilitates better entrapment of Rp, however with high risk of transformation into perfect Lβ arrangement upon storage, favoring faster and hazardous burst exclusion of the drug from the SLN matrix. Being Rp-SLN characterized by a consistent portion of Lβ polymorph, they may be less prone to change over time with respect to to blank SLN.

#### 2.1.6. *In Vitro* Drug Release Studies

Rp-SLN were characterized for *in vitro* release of the drug over 24 h by high performance liquid chromatography (HPLC) analysis to establish the possible fast release of the drug. Rp was not released from the SLN (<5%) over 24 h, confirming a stable entrapment within the lipid matrix (see Figure S1). The mass balance measurement supported further the retention of the drug inside the SLN.

### 2.2. Biological Characterization of Rp-SLN

#### 2.2.1. Cell Uptake

The efficacy of the Rp-SLN was evaluated in a well-established neuronal cell model, the human neuroblastoma-derived cell line SH-SY5Y [32].

Since Rp is a macrolide with a lactone ring moiety, it is prone to hydrolysis in solution. For this reason, the stability was checked in standard buffers and cell incubation media over time to ensure detectability of the drug during cell interaction studies (see Figure S1). According to the obtained data, the drug degradation was considered negligibile over 3–4 h in Dulbecco’s modified Eagle’s medium (DMEM) and DMEM supplemented with fetal bovine serum (FBS).

**Figure 7:**
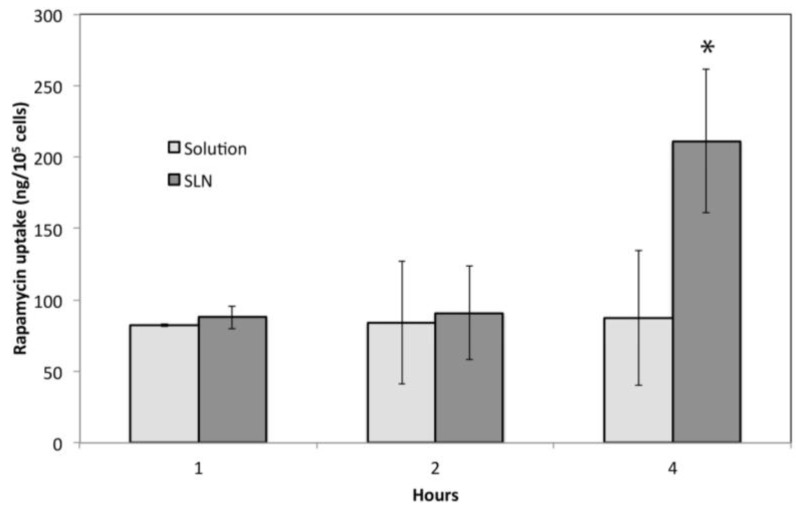
Cell uptake of SLN. Amount of Rp taken up by SH-SY5Y cells after 1, 2 and 4 h after the treatment with 200 nM Rp-SLN and Rp solution. * *p* < 0.001.

The kinetics of the uptake in SH-SY5Y cells observed for the Rp solution and the Rp-SLN dispersion, differed significantly. In fact, the 4 hour-long Rp-SLN treatment with Rp-SLN gives rise to two-fold drug accumulation within the cells (*p* < 0.001) with respect to free Rp (Figure 7). The study was not pushed further so prevent Rp from degradation in the medium and, taking into account the fact that the cell uptake likely occurrs through pinocytosis and endocytosis , a 4–6 h time frame is usually enough to allow the internalization. The higher amount of the drug internalized within Rp-SLN is expected as the Rp encapsulation into particles can concentrate the amount of the drug taken up per unit of time. The fact that up to 2 h no differences were observed in case of free Rp is likely related to the different uptake mechanism for the free drug and the particles, which conceivably may follow an endocytotic pathway for SLN and a faster micropinocytosis for Rp alone.

**Figure 8:**
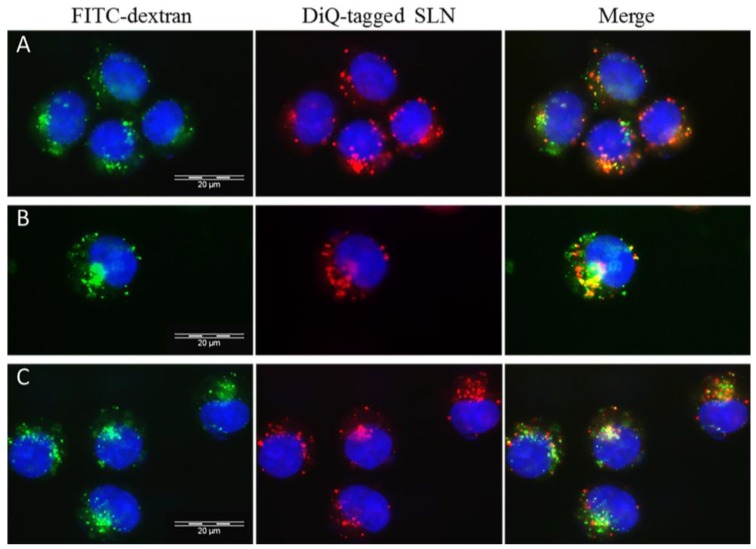
Cell uptake of fluorescent-SLN. Fluorescence microscopy images of SH-SY5Y cells were taken 1(**A**); 2 (**B**) and 4 h (**C**) after treatment with 500 nM DiQ-tagged Rp-SLN DiQ (red) and after staining of lysosomes with fluorescein isothiocyanate dextran (green). Nuclei were stained with 4',6-diamidino-2-phenylindole (DAPI). Magnification: 60×.

Fluorescent microscopy analysis, conduted for Rp-SLN tagged with a lipophilic dye DiQ, clearly confirmed the accumulation of Rp-SLN in the cells over time (Figure 8). The presence of Rp-SLN is evident after 1 h, however it increases at 4 h. The merged images show prevalent co-localization of SLN with lysosomes what confirms a dominant endocytotic mechanism for the Rp-SLN uptake.

Therefore, by using Rp-SLN formulation, the drug is delivered to lysosomes, and becomes available upon the lysosomal degradation of SLN. This may also produce a longer lasting effect for Rp-SLN by virtue of longer retention in cells, when compared to free Rp. This aspect was further investigated by pulse-chase study.

#### 2.2.2. Rp-SLN Have a Long Lasting Effect on Cell Proliferation

**Figure 9:**
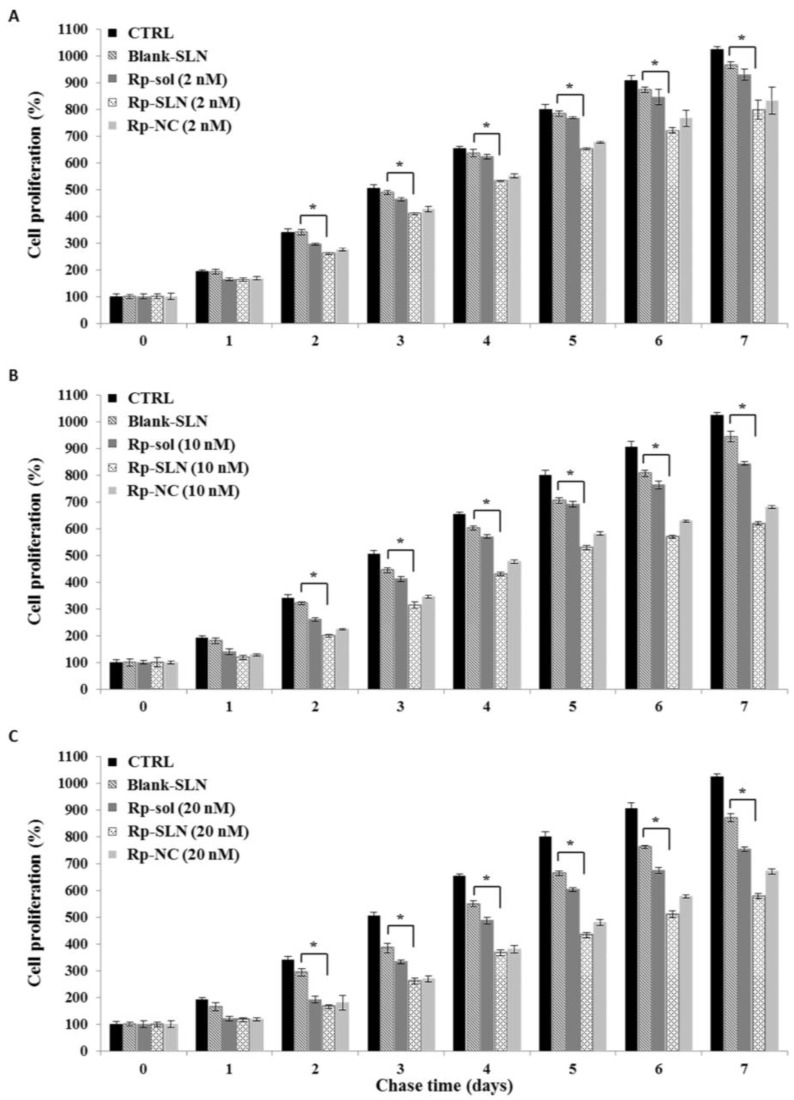
Effect of the Rp treatments on cell proliferation. The Rp effect was evaluated in SH-SY5Y cells by pulse-chase experiments. Cells were seeded in a 96-well plate, incubated overnight at 37 °C and treated for 4 h with Rp solution (Rp-sol), Rp-nanocrystals (Rp-NC) and Rp-SLN at the concentration of 2, 10 and 20 nM (Pulse), panel (**A**–**C**), respectively. The cells were also treated with blank SLN as control. The cell proliferation was evaluated daily by using 3-[4,5-dimethylthiazol-2-yl]-2,5-diphenyltetrazolium bromide (MTT) assay. Control (CTRL): untreated cells. Values are the mean ± S.D. of three independent experiments. * *p* < 0.01 (Rp-SLN *vs.* blank SLN cells) according to unpaired two-tailed Student’s *t*-test.

As it is known that Rp treatment affects cell proliferation [46], we compared the inhibitory effect of Rp-SLN, Rp-nanocrystals (Rp-NC), and Rp solution (Rp-sol) on proliferation of SH-SY5Y cells by means of pulse-chase experiments (Figure 9). The Rp-NC were used as a further control for non-encapsulated solid dispersion of Rp (see Figure S2). Once in the cell, although dispersed in the NC form, Rp is believed to be more readily available than that entrapped in the SLN. This should affect the duration of the impact on cells that is thought to be between that of the free Rp and Rp-SLN. Cells were also treated with blank SLN as a control. The SH-SY5Y cells were treated for 4 h with Rp-sol, Rp-NC and Rp-SLN at the concentration of 2, 10 and 20 nM (pulse), and cell proliferation was futher evaluated daily for 7 days (chase) by using 3-[4,5-dimethylthiazol-2-yl]-2,5-diphenyltetrazolium bromide (MTT) assay. As reported in Figure 9, all Rp formulations reduced the cell growth in a concentration-dependent manner. In particular, MTT data display a significantly lower level of cell proliferation with the Rp-SLN treatment when compared to Rp-sol and Rp-NC. Moreover, when the highest concentration of the drug was used, the decrease of cell proliferation resulted more persistent over time in the case of Rp-SLN, suggesting advantageous prolongation of the actual activity of Rp in a nanoparticles composite, when referred to the free Rp or NC formulations. Blank SLN did not affect the cell proliferation apart from a very low effect at higher concentrations. The effects of DMSO and PS80 on the cell proliferation were also evaluated and the results excluded any cytotoxic influences of the solvent and the surfactant (data not shown).

#### 2.2.3. Rp-SLN Show Prolonged Effect on mTOR Pathway

**Figure 10:**
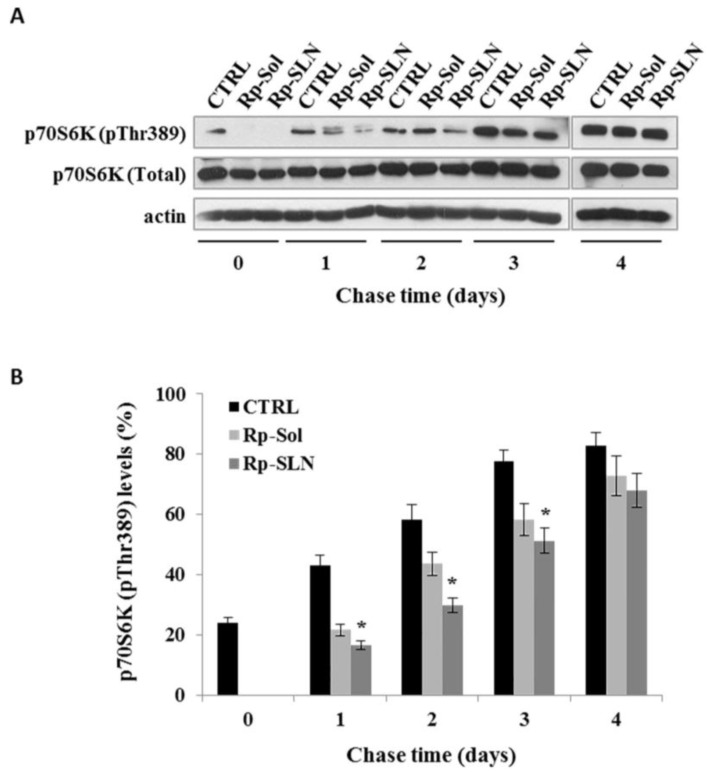
Effect of Rp-SLN on mammalian target of rapamycin (mTOR) activity. The SH-SY5Y cells were seeded in a 6-well plate, incubated overnight at 37 °C and then treated for 4 h with 20 nM of Rp-sol or Rp-SLN (Pulse); untreated cells were considered as control (CTRL). (**A**) Cells were recovered after 0, 1, 2, 3 and 4 days (chase) and the immunoblotting analysis was performed for phospho-p70S6K (pThr389), p70S6K (Total) and actin. Representative Western blotting of three independent experiments is reported; (**B**) densitometric analysis of the immunoblot represents the percentage of the ratio between phospho-p70S6K (pThr389) with respect to p70S6K. Values are the mean ± S.D. of three independent experiments. * *p* < 0.01 (Rp-SLN *vs.* CTRL cells) according to unpaired two tailed Student’s *t*-test.

The biological activity of Rp on the mTOR pathway was evaluated in SH-SY5Y cells by immunoblotting analysis, performed to verify the phosphorylation status of p70S6K, a substrate of mTORC1 [13]. Cells were treated for 4 h with 20 nM of both Rp-sol and Rp-SLN. Untreated cells were considered as control (CTRL). Cells were recovered upon 0, 1, 2, 3 and 4 days to conduct the immunoblotting analysis. As reported in Figure 10, both Rp-sol and Rp-SLN treatments inhibit the phosphorylation of p70S6K. However, densitometric analysis clearly shows that the Rp-SLN treatment is more effective in inhibition of the p70S6K phosphorylation (*p* < 0.01) (Figure 10B).

These results are in agreement with those presented for the cell proliferation and further demonstrate that Rp-SLN are unarguably more effective than free Rp.

## 3. Materials and Methods

### 3.1. Preparation of Solid Lipid Nanoparticles

Blank and Rp-SLN were prepared by using two different methods: (i) a cold High-Pressure Homogenization/evaporation (cHPHe) protocol; and (ii) an Ultrasound-Assisted Emulsion/evaporation method (UAEe).

#### 3.1.1. Cold High Pressure Homogenization/Evaporation Method

Briefly, Rp (NH Co. Ltd., Zhejiang, China) and cetyl palmitate or Compritol^®^ 888 ATO (Gettafosse, Milan, Italy), weighed/mixed in proportion 0:10 and 1:9 (*w*/*w*), for blank SLN and Rp-SLN, respectively, were dissolved properly in 2 mL of chloroform and transferred drop-wise into a glass tube containing 1% (*w*/*v*) PS80 solution (Sigma-Aldrich, Milan, Italy), stirred up continuously by an UltraTurrax homogenizer (IKA, Milan, Italy). The oil/water emulsion was subsequently processed in an EmulsiFlex-C5 high-pressure homogenizer (Avestin Inc, Ottawa, Canada), over 5 homogenization cycles at 1500 bars. Chloroform was then removed by evaporation.

#### 3.1.2. Ultrasound-Assisted Emulsion/Evaporation Method

The solutions were prepared as above described. The Rp chloroform solution was slowly added dropwise (0.2 mL/min) to the 1% *w*/*v* PS80 solution at room temperature (r.t.) under sonication. Sonication was continued until the complete organic solvent evaporation by controlling the temperature increase. At the end of the process the SLN dispersion was cooled down in an ice bath.

The SLN preparations were purified for non-encapsulated Rp and excess PS80 by using both dialysis for 48 h in 1 L of water in a 14 kDa dialysis bags and centrifugation (20 min at 4000 rpm) in 6 mL Vivaspin tubes (cut-off = 100 kDa, Amicon Ultra, Milan, Italy).

### 3.2. Preparation of Rapamycin Nanocrystals

Nanocrystals of Rp (Rp-NC) preparation was conducted using the same cHPHe method described above with proper modification. Rp-NC were used as solid dispersion of Rp to be compared in the cell study. Briefly, 30 mg of Rp were dissolved in 0.5 mL of chloroform and added drop-wise into 1% (*w*/*v*) PS80 aqueous solution upon mixing using an UltraTurrax homogenizer. Then, the emulsion was homogenized using the Emulsiflex-C5 high pressure homogenizer through 25 cycles at 1500 bars. Rp-NC were dialyzed for 48 h in 1 L of 1% PS80 aqueous solution and stored at 4 °C.

### 3.3. Drug Content

Drug content (%DC) in Rp-SLN samples was measured by using UV-Vis spectroscopy. Freshly prepared samples were placed in plastic tubes, diluted with distilled water and ultracentrifuged (100,000 rpm, 1 h at 4 °C; Optima TL100, Beckman, Milan, Italy). The supernatant, containing free Rp, was separated from the pellet and both supernatant and pellet were extracted in a chloroform-DMSO mixture. UV-Vis measurements were performed by an Agilent 8453 diode array UV-Vis spectrophotometer (Agilent, Milan, Italy), at λ_max_ = 278 nm using pure chloroform as a reference. Calibration was performed in chloroform in the 5–20 μg/mL concentration range (*r*^2^ = 0.999).

### 3.4. Dynamic Light Scattering and Zeta-Potential

Size distribution and zeta potential (Pz) measurements were performed using a Nicomp 380 ZLS autocorrelator (PSS Inc., Santa Barbara, CA, USA) equipped with a He-Ne Coherent Innova 70-3 (Laser Innovation, Moorpark, CA, USA) laser (λ = 658 nm, 45 mW) and an avalanche photodiode detector (APD) and a phase analysis of light scattering PALS system. Polydispersity (PD) of SLN size distribution and mean hydrodynamic diameter (MHD) were calculated on triplicate samples. Pz analysis was performed by applying 180 s cycle of 1 mV electric field and by acquiring the scattering intensity at 14.9 degrees scattering angle at 23 °C.

### 3.5. Particle Tracking

Particle tracking analysis for both blank and Rp-SLN was measured by means of a conventional NanoSight LM10 NTA system (Malvern Instruments Ltd, England). The instrument is equipped with a laser beam at 635 nm finely focused on a sample by a 20× objective and supplemented with a coupled-charge device (CCD)-camera recording data at 30 frames/s. Size distributions were calculated on particles traveling on a *XY* plane at the minimal track length value of 50 (a. u.). Measurements were carried out at 25, 37 and 50 °C.

### 3.6. Cryo-Transmission Electron Microscopy

Morphology and structure quality of SLN were investigated by means of cryo-transmission electron microscopy (cryo-TEM). Blank and Rp-SLN preparations were layered on a Lacey Formvar/Carbon-coated copper grid of 300 mesh (TedPella, Inc., Redding, CA, USA), immediately frozen in liquid nitrogen and mounted in the cryo-chamber of the TEM microscope (JEM-1400, JEOL USA Inc., Peabody, MA, USA). Images were acquired by applying the maximum acceleration voltage of 120 kV.

### 3.7. Wide-Angle X-ray Scattering

Wide-angle X-ray scattering (WAXS) studies of pure Rp powder, blank SLN and Rp-SLN were carried out on an Empyrean (PANalytical) diffractometer (PANalytical Srl, Milan, Italy) using Cu Kα radiation (1.54 Å), reflection-transmission spinner (sample stage) and PIXcel 3D detector, operating in the Bragg-Brentano geometry. The 2θ scans were recorded at room temperature in the 2θ range of 3 to 40 degrees with a step size of 0.006 degree and continuous scan mode. The repeat distance, *d* was calculated from the Bragg’s equations:

(1) $$q=\;\frac{4\pi \sin\left(\theta \right)}{\lambda }$$

with

(2) $$d=\frac{q}{2\pi }$$

where *q* is the scattering vector, θ the scattering angle and λ the wavelength of the source [47].

### 3.8. Differential Scanning Calorimetry

The DSC measurements were performed using a DSC-204 Phoenix Netzsch calorimeter equipped with a high sensitivity u-sensor. The calorimetric system was calibrated by using cyclohexane, mercury, indium, bismuth, tin and biphenyl. The samples of about 3–4 mg were sealed in hermetic aluminum crucibles. All measurements were performed in helium atmosphere between 30 and 80 °C at a scanning rate of 5 °C/min. Each sample was scanned successively two times using an empty crucible as a reference sample. The DSC data were processed and analyzed using the TA (Neztsch) program. For determination of melting enthalpies (ΔH_m_) and recrystallization enthalpies (ΔH_c_), a linear baseline was used. The recrystallization index RI% for blank and loaded SLN was calculated from Equation (1):

(3) $$RI\left(\%\right)=\;\frac{Enthalpy_{SLN}}{Enthalpy_{bulk\;material}\times Theoretical\;concentration_{lipid\;phase}(\%)}\times 100$$

Supercooling effect (SC) has been evaluated as the arithmetic difference between the endothermic melting peak and the exothermic recrystallization temperature [44].

### 3.9. Nuclear Magnetic Resonance Measurements

For NMR analysis both water suspensions of blank and Rp-loaded SLN were centrifuged in Amicon Viva Spin molecular weight cut-off = 100 kDa, 4000 rpm, 20 min (Amicon Ultra, Milan, Italy). Residual water was removed under nitrogen gas, and the samples were dispersed in 0.6 mL D_2_O (including a tetramethylsilane (TMS) standard, Sigma-Aldrich, Milan, Italy). The PS80 sample was prepared directly in D_2_O (1% *w*/*v*), whilst Rp sample (at concentration of 0.5 mg/mL) was prepared in DMSO-d_6_ (Sigma-Aldrich, Milan, Italy). All four samples were placed into 5 mm NMR tubes, and measured separately. ^1^H NMR experiments were performed at 25 °C on Agilent DD2 800 MHz NMR spectrometer (Agilent, Milan, Italy) operating at 18.8 T (1H resonance frequency: 799.903 MHz) equipped with a z-axis gradient unit and 5 mm ^1^H/^13^C/^15^N probe head. A residual HOD peak was suppressed with presaturation technique. To obtain satisfactory signal-to-noise ratio about 2k FID (free induction decay) were averaged. The spectral data were collected as 38k hyper complex data points with a spectral width 7622 Hz. After Fourier transform and phase correction all spectra were imported into Origin software (OriginLab Corp., Northampton, MA, USA) for further analysis.

### 3.10. In Vitro Drug Release from SLN

In order to determine Rp release from SLN in physiologic environment, indicative of the stability in the body circulation, *in vitro* studies were conducted by incubating 2 mL Rp-SLN suspension, purified from free Rp through centrifugation in Vivaspin tubes (Sartorius, Milan, Italy), at 37 °C loaded in a dialysis bag (molecular weight cut-off = 14 kDa) in 15 mL 10% (*v*/*v*) ethanol/ 0.1 M pH 7.4 phosphate buffer. A Rp solution at equivalent concentration of the SLN suspension was used as control. Aliquots were withdrawn at 2, 4, 6 and 24 h for SLN and 20, 40, 60 min, 4, 6 and 24 h for the control. At 24 h, the SLN were recovered by centrifugation to determine residual Rp. All measurements were performed in triplicate and the error expressed as S.D.

Rp was quantified by HPLC in the following conditions: eluent 60:40 acetonitrile:water, Zorbax SB300-C18 column equilibrated at 55 °C, flowrate 1 mL/min, UV-vis detector set at λ_max_ = 278 nm. Calibration was performed in the range 0.1–1 μg/mL (*r*^2^ = 0.99955).

### 3.11. Cell Culture

The SH-SY5Y neuroblastoma cell line was obtained from American Type Culture Collection (Manassas, VA, USA) and cultured in Dulbecco’s modified Eagle’s medium (DMEM) supplemented with 10% (*v*/*v*) heat-inactivated fetal bovine serum (FBS), 2 mM l-glutamine, 100 *U* penicillin, 100 *U* streptomycin in a humidified incubator under 5% CO_2_ at 37 °C. The viability of the cell was verified by the standard Trypan Blue method.

### 3.12. Stability of Rapamycin in Cell Media

Stability of Rp in a cell culture medium (DMEM) was investigated using the HPLC method described above. Stability tests were performed as follows: Rp (1 mg) was incubated in 50 mL of DMEM, DMEM with antibiotics (1%), DMEM with serum (10%) and DMEM with both antibiotics and serum at 37 °C, and analyzed by HPLC up to 24 h. The hydrolysis was estimated measuring decay of the Rp peak area. All measurements were performed in triplicate and expressed as mean ± S.D.

### 3.13. Cell Uptake

SH-SY5Y cells were seeded in flasks at a concentration of 50 × 10^3^ cells/cm^2^ and incubated overnight at 37 °C under 5% CO_2_. Subsequently, the cells were washed with D-PBS and treated for 1, 2 and 4 h with 1% DMSO-water Rp-sol or Rp-SLN suspension, both at 200 nM Rp concentration. After each period of incubation, cells and medium were recovered and analyzed by HPLC. Data were normalized to cell number in each flask.

### 3.14. Fluorescent Microscopy

The SH-SY5Y cells were plated onto glass coverslips and incubated for 24 h at 37 °C under 5% CO_2_. Cells were treated for 2 h with 500 nM fluorescent Rp-SLN suspension and 2 mg/mL of FITC-dextran (Sigma-Aldrich). The SLN were labeled with DiQ (Invitrogen, Milan ,Italy) by adding the tag to the SLN suspension (5 μL/mL). After 10 min, the suspension was purified by centrifugation (4000 rpm, 15 min, 4 °C) in Vivaspin tubes to remove free tag and excess PS80.

At the end of each time period, the cells were fixed with 4% (*v*/*v*) paraformaldehyde for 30 min at room temperature and washed three times with phosphate-buffer saline (PBS). Coverslips were mounted on glass slides using Vectashield with DAPI (Vector Laboratories Inc., Burlingame, CA, USA). Fluorescence microscopy analysis was performed using a Nikon TE2000 microscope (Nikon Instruments S.p.A, Florence, Italy) through a 60× oil immersion objective. Image processing was performed using Adobe-Photoshop CS software (Adobe Systems Incorporated).

### 3.15. Pulse-Chase Studies

SH-SY5Y cells were seeded in a 96-well plate (1.6 × 10^4^ cells/well) and incubated overnight at 37 °C under 5% CO_2_. Then, the cells were washed with D-PBS and treated for 4 h with Rp-sol, Rp-NC and Rp-SLN at concentration of 2, 10 and 20 nM (Pulse). As control, cells were also treated with equivalent concentration of DMSO used to resuspend free Rp, 1% (*w*/*v*) PS80 used for both Rp-NC and Rp-SLN and with blank SLN. Treated cells were chased for 7 days. Cell proliferation was evaluated daily by using 3-[4,5-dimethylthiazol-2-yl]-2,5-diphenyltetrazolium bromide (MTT) assay according to the manufacturer’s manual. Briefly, 10 μM of MTT was added in each well and the cells were incubated for 4 h at 37 °C. Successively, 100 µL of solubilization solution (10% sodium dodecyl sulfate (SDS) with 0.01 N HCl) was added to each well and solubilization of formazan crystals was performed overnight at 37 °C. Cell proliferation was assessed spectrophotometrically at 570 nm with 650 nm reference wavelength on Beckman Coulter DTX 880 Multimode Detector (Beckman Coulter Inc., Brea, CA, USA).

### 3.16. SDS-PAGE and Western Blotting

SH-SY5Y cells were seeded in 6-well plate (50 × 10^3^ cells/cm^2^) and incubated overnight at 37 °C under 5% CO_2_. Then, the cells were washed with D-PBS and treated for 4 h with 20 nM of both Rp-Sol and Rp-SLN (Pulse). Untreated cells were considered as control. Chased cells were recovered after 0, 1, 2, 3 and 4 days and resuspended in lysis buffer (50 mM Tris-HCl pH 7.2 with 0.5% (*v*/*v*) Triton X-100) containing protease and phosphatase inhibitors (Sigma-Aldrich). After 30 min of incubation at 4°C, the samples were centrifuged at 16,000× *g* for 15 min at 4 °C. Supernatant was recovered and considered as cell extract. Total protein concentration was determined by Bradford assay using bovine serum albumin as a standard [48]. Proteins (50 µg/lane) were separated by 10% SDS-PAGE under reducing conditions (Mini-Protean III, Biorad, (Bio-Rad Laboratories S.r.l., Milan, Italy) according to a Laemmli protocol [49] and, successively electrotransferred to a PVDF membrane (Mini Trans-Blot Cell, Bio-Rad Laboratories S.r.l., Milan, Italy). Detection of the proteins was performed by overnight incubation with primary antibodies as follow: 1:1000 of rabbit polyclonal anti-Phospho-p70S6K (Thr389) (Cell Signaling Technology Inc., Danvers, MA, USA), 1:1000 of rabbit polyclonal anti-p70S6K (Cell Signaling Technology Inc) and 1:5000 of monoclonal anti-actin (Sigma-Aldrich). After being washed using TBS containing 0.1% (*v*/*v*) Tween 20, the membrane was incubated with secondary antibodies as necessary: anti-rabbit IgG (Cell Signaling Technology Inc) or anti-mouse IgG (GE Healthcare) horseradish peroxidase (HRP)-conjugated. Detection was performed by using ECL detection system (GE Healthcare) according to the manufacturer’s manual.

### 3.17. Statistical Analysis

Statistical analysis of the cell study data was performed by unpaired two tailed Student’s *t*-test and ANOVA.

## 4. Conclusions

The compritol Rp-SLN obtained by the UAEe method seem to possess promising features in terms of inner structure, morphology, and short-term stability. The lack of immediate release is potentially useful to prevent drug losses before reaching the target in the organism. PS80 seems to be stably anchored to the particle surface and Rp is entrapped inside the lipid core, which is important for two reasons: (i) avoiding unwanted release of the drug; and (ii) avoiding possible hindrance preventing the binding of PS80 to apolipoproteins, which is essential to grant brain uptake. The biological characterization, based on the well known Rp activity on the mTOR pathway, demonstrated that entrapment in SLN improves time activity profile of the drug, allowing an extended effect and enhanced efficacy on cell proliferation decrease and p70S6K phosphorylation inhibition. Overall, Rp-SLN can be considered worth of being further investigated as possible brain targeting system for Rp in order to reduce the adverse systemic immunosuppression typical of Rp treatment.

## Figures and Tables

**Table 1:** Characterization of Rp loaded solid lipid nanoparticles (Rp-SLN) and blank SLN obtained by the Ultrasound-Assisted Emulsion/evaporation (UAEe) and the cold High Pressure Homogenization/evaporation (cHPHe) methods.

**Ultrasound-Assisted Emulsion/Evaporation Method**							
**Batch No.**	**Working Conditions**	**Drug Loading (% *w*/*w*)**	**Lipid**	**Gaussian MHD (nm, PI)**	**Pz (mV)**	**%DC (*w*/*w*)** **±** **S.D.**	**%EE** **±** **S.D.**
#1	2% *w*/*v* PS80, 1.5 mL CHCl_3_, vortex, r.t.	0	Cetyl palmitate	249 (0.371)	−5	-	-
#2	2% *w*/*v* PS80, 1.5 mL CHCl_3_, vortex, r.t.	10	Compritol	102 (0.531)	−16	4.7 ± 1.1	43.2 ± 0.1
#3	2% *w*/*v* PS80, 1.5 mL CHCl_3_, vortex, r.t.	10	Cetyl palmitate	70 (0.349)	−13	2.2 ± 0.8	21.5 ± 0.1
#4	2% *w*/*v* PS80, 1.5 mL CHCl_3_, manual agitation, r.t.	10	Compritol	204 (0.594)	−5	4.1 ± 1.1	41.4 ± 0.1
#5	2% *w*/*v* PS80, 1 mL CHCl_3_, Ti = 42 °C	10	Cetyl palmitate	265 (0.311)	−8	0.5 ± 0.1	2.6 ± 0.5
#6	2% *w*/*v* PS80, 1 mL CHCl_3_, Ti = 42 °C	10	Cetyl palmitate	93 (0.301)	−7	0.6 ± 0.1	6.3 ± 0.2
#7	1% *w*/*v* PS80, 1 mL CHCl_3_, Ti = 42 °C	10	Compritol	267 (0.497)	−11	4.4 ± 0.5	43.8 ± 0.9
#8	1% *w*/*v* PS80, 1 mL CHCl_3_, Ti = 42 °C	0	Compritol	244 (0.401)	−12	-	-
#9	1% *w*/*v* PS80, 1 mL CHCl_3_, Ti = 42 °C	10	Cetyl palmitate	aggregates	−16	2.3 ± 0.2	23.2 ± 0.9
#10	1% *w*/*v* PS80, 1 mL CHCl_3_, Ti = 42 °C	10	Cetyl palmitate	aggregates	−14	3.2 ± 0.2	32.2 ± 2.1
#11	1% *w*/*v* PS80, 1 mL CHCl_3_, Ti = 42 °C	10	Compritol	122 (0.303)	−12	4.1 ± 0.9	40.6 ± 4.2
#12	1% *w*/*v* PS80, 1 mL CHCl_3_, Ti = 42 °C	10	Compritol	186 (0.301)	−8	3.8 ± 0.7	37.5 ± 2.3
**Cold High Pressure Homogenization/Evaporation Method**							
**Batch No.**	**Working Conditions (5 Cycles, 1500 Bars)**	**Drug Loading (% *w*/*w*)**	**Lipid**	**Gaussian MHD (nm, PI)**	**Pz (mV)**	**%DC (*w*/*w*)** **±** **S.D.**	**%EE** **±** **S.D.**
#1	2 mL CHCl_3_, 1% *w*/*v* PS80, r.t. evaporation	0	Cetyl palmitate	225 (0.284)	−3	-	-
#2	2 mL CHCl_3_, 1% *w*/*v* PS80, r.t. evaporation	20	Cetyl palmitate	296 (0.548)	−20	2.9 ± 0.6	14.5 ± 0.1
#3	2 mL CHCl_3_, 1% *w*/*v* PS80, r.t. evaporation	10	Cetyl palmitate	212 (0.397)	−10	8.9 ± 1.7	89.3 ± 2.5
#4	2 mL CHCl_3_, 1% *w*/*v* PS80, rotavapor	10	Cetyl palmitate	237 (0.462)	−2	7.4 ± 0.9	73.8 ± 2.6
#5	2 mL CHCl_3_, 1% *w*/*v* PS80, rotavapor	20	Cetyl palmitate	123 (0.442)	−1	16.8 ± 3.1	84.0 ± 5.4
#6	2 mL CHCl_3_, 1.5% *w*/*v* PS80, rotavapor	10	Cetyl palmitate	375 (0.666)	1	5.4 ± 0.8	54.3 ± 6.5
#7	2 mL CHCl_3_, 1% *w*/*v* PS80, rotavapor	0	Compritol	267 (0.497)	−12	-	-
#8	2 mL CHCl_3_, 1% *w*/*v* PS80, rotavapor	10	Compritol	aggregates	−1	7.7 ± 1.4	77.0 ± 5.4
#9	2 mL CHCl_3_, 1% *w*/*v* PS80, rotavapor	20	Compritol	aggregates	−1	8.9 ± 1.3	44.5 ± 4.2
#10	1.5 mL CHCl_3_, 1% *w*/*v* PS80, rotavapor	10	Compritol	751 (0.812)	−20	4.4 ± 0.7	43.8 ± 3.5

r.t.: room temperature; MHD: mean hydrodynamic diameter; PI: polydispersity index; Pz: zeta potential; DC: drug content; S.D.: standard deviation; EE: encapsulation efficiency.

**Table 2:** Thermodynamic parameters measured for blank and Rp-SLN in comparison with bulk compritol. Data expressed as mean ± S.D. RI: recrystallization index; SC: supercooling effect.

Sample	Thermal process	ΔH (J/g)	Peaks (°C)		T_Onset_ (°C)	T_End_ (°C)	RI (%)	SC (°C)
			Major	Minor				
**Compritol**	**Heating**	120.6 ± 2.6	69.7 ± 1.7	67.8 ± 2.1	66.3 ± 1.5	70.4 ± 1.6	100	5.1 ± 0.4
	**Cooling**	96.4 ± 2.1	64.2 ± 1.2	63 ± 0.7	62.4 ± 1.7	65.1 ± 1.7		
**Blank SLN**	**Heating**	39.7 ± 0.8	66.5 ± 1.1	64.7 ± 1.4	63.6 ± 1.7	69.6 ± 0.5	33.3 ± 1.4	3.6 ± 0.2
	**Cooling**	30.5 ± 0.9	63.3 ± 1.6	62.7 ± 1.6	62.0 ± 0.9	64.4 ± 0.8		
**Rp-SLN**	**Heating**	48.5 ± 1.2	63.9 ± 1.5	62.9 ± 0.9	61.8 ± 0.6	66.7 ± 1.3	40.5 ± 1.9	0.8 ± 0.1
	**Cooling**	40.9 ± 0.7	63.0 ± 0.8	61.8 ± 1.4	61.6 ± 1.1	63.6 ± 1.3		

**Table 3:** Wide angle X-ray scattering (WAXS) main peak signals and Bragg’s lattice spacings for blank and Rp-SLN in comparison with bulk compritol.

Compritol		Blank SLN		Rp-SLN	
2θ	*d* (nm)	2θ	*d* (nm)	2θ	*d* (nm)
4.20	2.102	4.36	2.023	4.35	2.029
5.58	1.582	5.71	1.546	5.57	1.585
7.07	1.248	7.17	1.207	7.30	1.209
8.41	1.023	8.67	1.027	8.67	1.026
11.22	0.786	11.57	0.757	11.51	0.768
-	-	19.48	0.455	19.30	0.459
21.14	0.420	21.35	0.416	21.38	0.415
23.25	0.382	23.41	0.380	23.60	0.376

## Supplementary Materials

the following are available online at http://www.mdpi.com/2079-4991/6/5/87/s1.
