# Supplementary material for: Rapamycin Loaded Solid Lipid Nanoparticles as a New Tool to Deliver mTOR Inhibitors: Formulation and in Vitro Characterization
Source: Nanomaterials (Basel). 2016 May 9;6(5):87. doi: 10.3390/nano6050087 (PMC5302501; doi:10.3390/nano6050087)
Supplement: Supplementary file 1 [file nanomaterials-06-00087-s001.pdf]

Supplementary Material: Rapamycin Loaded Solid Lipid Nanoparticles as a New Tool to Deliver mTOR Inhibitors: Formulation and *in Vitro* Characterization

Alice Polchi, Alessandro Magini, Jarosław Mazuryk, Brunella Tancini, Jacek Gapiński, Adam Patkowski, Stefano Giovagnoli and Carla Emiliani

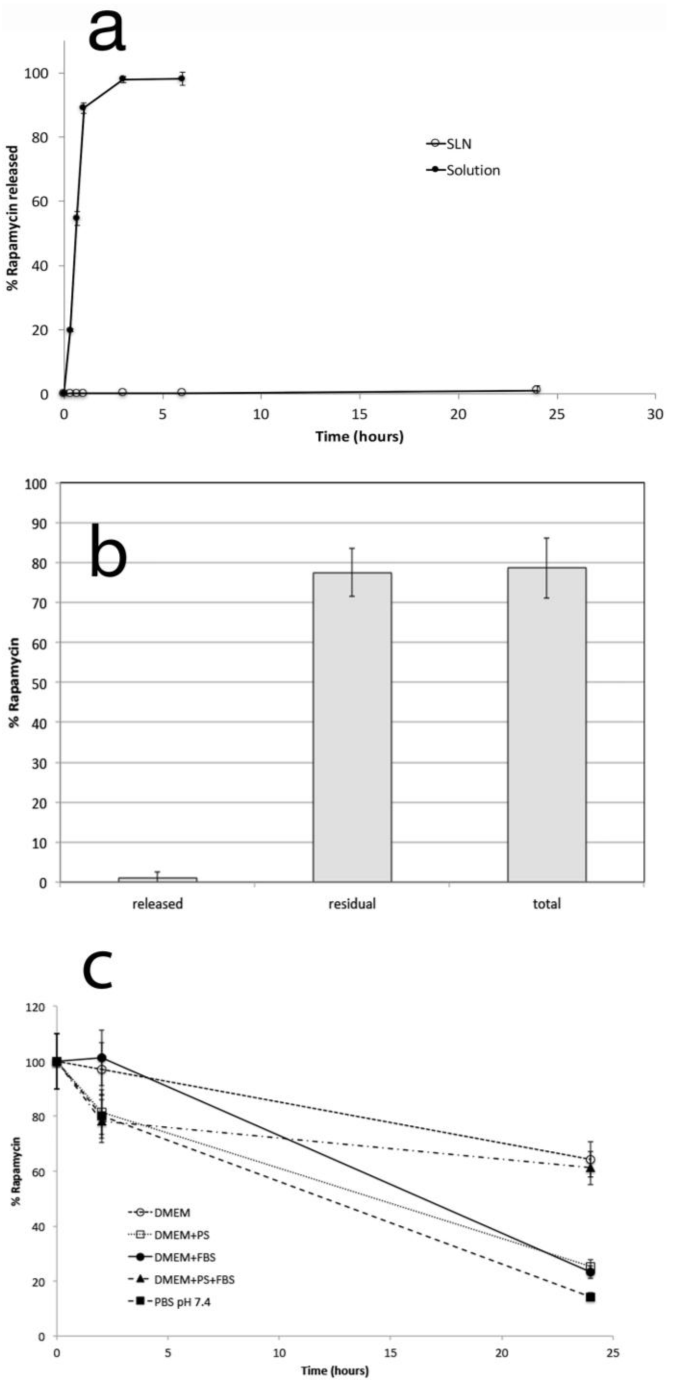

Figure S1. Rapamycin (Rp) stability in standard buffers and culture medium. (**a**) *In vitro* Rp release from Rp loaded solid lipid nanoparticles (Rp-SLN) in 10% *v*/*v* ethanol: 0.1 M pH 7.4 phosphate buffer over 24 h at 37 °C; (**b**) Rp mass balance after 24 h between the released amount and the amount retained inside the SLN; (**c**) stability profile of Rp in different media over 24 h at 37 °C

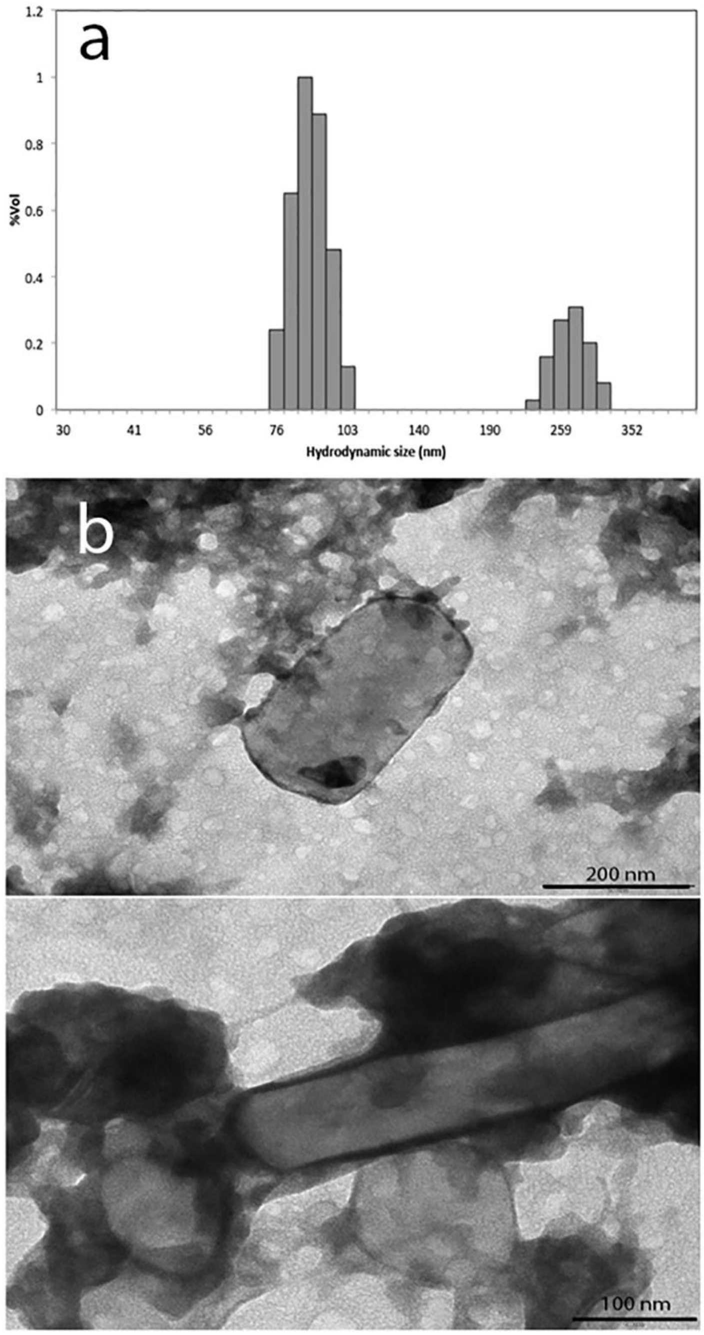

Figure S2. Rp nanocrystals characterization**.** (**a**) Size distribution of Rp nanocrystals; (**b**) Transmission electron microscopy (TEM) images of Rp nanocrystals, magnification 180,000×.
